# Maximum blink interval is associated with tear film breakup time: A new simple, screening test for dry eye disease

**DOI:** 10.1038/s41598-018-31814-7

**Published:** 2018-09-07

**Authors:** Takenori Inomata, Masao Iwagami, Yoshimune Hiratsuka, Keiichi Fujimoto, Yuichi Okumura, Tina Shiang, Akira Murakami

**Affiliations:** 1 Juntendo University Faculty of Medicine, Department of Ophthalmology, Tokyo, Japan; 2 Juntendo University Faculty of Medicine, Department of Strategic Operating Room Management and Improvement, Tokyo, Japan; 3 London School of Hygiene and Tropical Medicine, Department of Non-Communicable Disease Epidemiology, London, UK; 4 University of Massachusetts Medical School, Worcester, MA USA

## Abstract

The prevalence of dry eye disease (DED) is increasing worldwide, and its diagnosis often needs dedicated reagents and machines. We investigated the usefulness of maximum blink interval (MBI) (the length of time that participants could keep their eyes open) in screening for DED. This cross-sectional study included 292 patients (194 with DED and 98 without DED) recruited between September 2016 and September 2017. We compared the MBI between patients with and without DED; examined correlations between MBI and other clinical features of DED, including subjective symptoms (Dry Eye-Related Quality-of-Life Score), tear film breakup time (TFBUT), cornea fluorescence score (CFS), and Schirmer test I value; and determined the optimal cutoff value of MBI to suspect DED using a receiver operating characteristic (ROC) analysis. The MBI was significantly shortened in DED group compared to the non-DED group (10.0 ± 9.1 vs. 24.3 ± 38.2 seconds, p < 0.001). TFBUT was strongly positively correlated with MBI (r = 0.464), whereas CFS was negatively correlated with MBI (r = −0.273). The area under the ROC curve was 0.677, and the optimal MBI cutoff value was 12.4 seconds, providing a sensitivity of 82.5% and specificity of 51.0% to suspect DED. In conclusion, MBI may be a simple, useful test for screening DED.

## Introduction

Dry eye disease (DED), a disorder of the tear film due to tear deficiency or excessive tear evaporation, damages the interpalpebral ocular surface and is associated with symptoms of ocular discomfort^1^. DED is diagnosed by triaging questions; risk factor analysis; diagnostic tests, such as questionnaire, non-invasive tear film breakup time (TFBUT), osmolality measurements and ocular surface staining^2^. Subsequently, subtype classification is performed using the Schirmer test and meibomian gland examination^2^. The prevalence of DED is currently estimated to be between 5 to 50% and will increase due to aging society^3^, environmental factors^4^, stressful social environments^5^, and increased digital device usage^4^. Since it is thought that there are many people with undiagnosed DED who have a decreased quality of life (and quality of vision)^6,7^ and work productivity^4^, it is important to develop a simple self-screening tool for DED that can trigger a consultation between a patient and an ophthalmologist. The clinical assessment of DED needs dedicated reagents and machines, these assessments tools are not suitable for self-screening.

Blinking is an essential function of the eye that helps to spread tears, mucin, and lipids on the cornea and conjunctiva, which maintain the eye’s moisture and protect the eye from irritants^8–10^. There are three types of blinking: voluntary blinking is a brief lid closure that is intended to maintain the visual activity; periodic blinking occurs continuously and unconsciously during the awake time; and reflex blinking occurs as a reflex to protect the eye^11^. Periodic blinking rate can be affected by various factors, such as fatigue, eye injury, medication, diseases, and dryness^10,12,13^.

Blink rates, interblink interval (IBI), and maximum blink interval (MBI) have been demonstrated to be useful in distinguishing between normal participants and patients with DED^14–17^ because the local ocular conditions has been shown to affect the pattern of blinking^17,18^. Currently, the instability of the tear film is considered to be one of the core mechanisms of DED^19,20^. Therefore, it has been hypothesized that measurement of MBI, which is a number of seconds the eyes can stay open without blinking^17^, can be simple a method to assess for DED. However, there have been few studies to assess the usefulness of MBI in the assessment of DED and to study the relationship between MBI and other clinical features of DED^15,17,21^.

In this study, we aimed to assess the usefulness of MBI in screening for DED by (i) comparing MBI between non-DED and DED patients, (ii) examining correlations between MBI and other test results for DED assessment, and (iii) conducting a receiver operating characteristic (ROC) analysis to identify the optimal MBI cutoff value to suspect DED. In addition, we assessed the blink interval period (BIP), which is the difference between MBI and TFBUT, to explore the mechanism of MBI.

## Results

### Participants’ characteristics

We enrolled 292 participants in this study. Table 1 shows the patients’ characteristics. All participants underwent complete examination and were eligible for analysis. The average age was 62.3 ± 14.9 years, and 82.2% of the participants were women. Based on the Asia Dry Eye Society (ADES) diagnostic criteria^19^, 98 participants were diagnosed as non-DED (33.6%), whereas 194 participants were diagnosed to have DED (66.4%). Positive subjective symptom rates, the Dry Eye-Related Quality-of-Life Score (DEQS)^22^, and corneal fluorescein staining (CFS) scores were significantly higher in the DED patients than in the non-DED participants. TFBUT was significantly lower in the DED patients than in the non-DED participants. The Schirmer test value was not significantly different between the groups.

**Table 1:** The characteristics of study participants.

Classification Characteristics	Non-DED n = 98	DED n = 194	P value	Total N = 292
Age, y ± SD	65.6 ± 15.2	60.6 ± 14.6	0.007	62.3 ± 14.9
Female, n (%)	71 (72.4)	169 (87.1)	0.002	240 (82.2)
BCVA, LogMAR ± SD	0.0 ± 0.2	0.0 ± 0.2	0.043	0.0 ± 0.2
IOP, mmHg ± SD	13.2 ± 2.4	14.0 ± 2.8	0.028	13.7 ± 2.7
Subjective symptom, yes (%)	15 (15.3)	194 (100.0)	<0.001	209 (71.6)
DEQS, 0–100 ± SD	11.6 ± 13.5	36.1 ± 22.4	<0.001	27.8 ± 23.0
TFBUT, sec ± SD	3.2 ± 3.4	1.6 ± 1.0	<0.001	2.1 ± 2.3
CFS score, 0–9 ± SD	2.4 ± 2.0	3.4 ± 2.3	<0.001	3.1 ± 2.3
Schirmer Ι, mm ± SD	6.0 ± 5.5	5.2 ± 6.2	0.280	5.5 ± 6.0
MBI, sec ± SD	14.4 ± 8.9	9.5 ± 6.5	<0.001	11.1 ± 7.7
BIP, sec ± SD	11.2 ± 7.7	8.4 ± 9.0	<0.001	9.0 ± 8.9

P values were estimated using the *t* test for continuous variables and χ^2^ test for categorical variables. DED; dry eye disease, BCVA; best-corrected visual acuity, IOP; intraocular pressure, DEQS; the Dry Eye-Related Quality-of-Life Score, TFBUT; tear film breakup time, CFS; corneal fluorescein staining, MBI; maximum blink interval, BIP; blink induction period.

### Maximum blink interval (MBI) and blink induction period (BIP) between non-DED and DED

MBI and BIP were compared between the non-DED and DED groups (Table 1). MBI was significantly shorter in the DED group than in the non-DED group (9.5 ± 6.5 vs. 14.4 ± 8.9 seconds, p < 0.001). BIP was also significantly shorter in the DED group than in non-DED group (8.4 ± 9.0 vs. 11.2 ± 7.7 seconds, p < 0.001).

### Correlations between MBI and other clinical assessment findings in DED

We examined the relationship between MBI and other clinical assessment findings in DED (DEQS, TFBUT, CFS and Schirmer test Ι) using the Pearson correlation test (Table 2). MBI was strongly correlated positively with TFBUT (r = 0.464) and negatively correlated with CFS (r = −0.273).

**Table 2:** The correlations between maximum blink interval and other clinical findings in dry eye disease.

Clinical items	MBI	DEQS	TFBUT	CFS	Schirmer test I
MBI	1.000				
DEQS	*−0.168	1.000			
TFBUT	*0.464	−0.106	1.000		
CFS	*−0.273	0.015	*−0.298	1.000	
Schirmer test I	0.105	0.028	*0.188	*−0.215	1.000

Pearson correlation coefficients were estimated among MBI, DEQS, TFBUT, and Schirmer test Ι. MBI; maximum blink interval, DEQS; the Dry Eye-Related Quality-of-Life Score, TFBUT; tear film breakup time, CFS; corneal fluorescein staining. P values are considered statistically significant at *p < 0.05.

### The cutoff value of MBI for detecting DED

**Figure 1:**
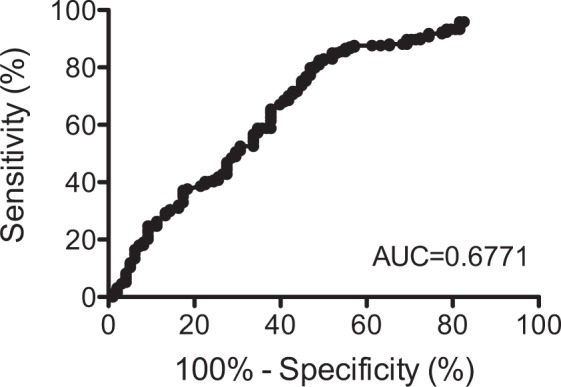
The receiver operator characteristic (ROC) curve for the detection of dry eye disease (DED) using maximum blink interval (MBI). (**A**) Represents the ROC curve for the detection of non-DED or DED group classified by the Asia Dry Eye Society diagnostic criteria using maximum blink interval (MBI). The AUC is 0.677 (95% CI: 0.610–0.744). ROC; receiver operator characteristic curve, DED; dry eye disease, MBI; maximum blink interval, AUC; area under the ROC curve.

Figure 1 shows the ROC curve illustrating the balance between sensitivity and specificity of different values of MBI in suspecting DED. The area under the ROC curve (AUC) was 0.677 (95%CI: 0.610–0.744). The optimum MBI cutoff value was 12.4 seconds, providing the sensitivity of 82.5% and the specificity of 51.0%.

### The precision rate and characteristics of participants detected by MBI at the cutoff value of 12.4 seconds

Table 3 shows the precision rate under the cutoff MBI value of 12.4 seconds. The positive predictive value was 76.9% (160/208), and the negative predictive value was 59.5% (50/84). Table 4 shows the characteristics of participants divided into groups based on the MBI cutoff value of 12.4 second. Positive subjective symptoms rate, DEQS, TFBUT, CFS, and BIP were significantly worse in the less-than-12.4-seconds MBI group compared to more-than-12.4-seconds MBI group.

**Table 3:** The precision rate detected by maximum blink interval at the cutoff value of 12.4 seconds.

Precision rate	Non-DED	DED	Total
MBI > 12.4 seconds (%)	50 (51.0)	34 (17.5)	84 (28.8)
MBI ≤ 12.4 seconds (%)	48 (49.0)	160 (82.5)	208 (71.2)
Total	98 (100)	194 (100)	292 (100)

MBI; maximum blink interval, DED; dry eye disease.

**Table 4:** The characteristics of participants detected by maximum blink interval at the cutoff value of 12.4 seconds.

Characteristics	MBI > 12.4 n = 84 (28.8)	MBI ≤ 12.4 n = 208 (71.2)	P value
Age, y ± SD	63.3 ± 14.2	61.9 ± 15.2	0.480
Female, n (%)	62 (73.8)	178 (85.6)	0.017
BCVA, LogMAR ± SD	0.0 ± 0.1	0.0 ± 0.2	0.320
IOP, mmHg ± SD	14.2 ± 2.9	13.5 ± 2.6	0.058
Subjective symptom, yes (%)	46 (54.8)	163 (78.4)	<0.001
DEQS, 0–100 ± SD	22.6 ± 21.3	30.1 ± 23.3	0.013
TFBUT, sec ± SD	3.6 ± 3.3	1.5 ± 1.3	<0.001
CFS score, 0–9 ± SD	2.1 ± 1.9	3.5 ± 2.3	<0.001
Schirmer Ι, mm ± SD	6.4 ± 6.6	5.1 ± 5.6	0.080
BIP, sec ± SD	17.6 ± 6.9	5.5 ± 2.6	<0.001

P values were determined using the *t* test for continuous variables and χ^2^ test for categorical variables. MBI; maximum blink interval, BCVA; best-corrected visual acuity, IOP; intraocular pressure, DEQS; the Dry Eye-Related Quality-of-Life Score, TFBUT; tear film breakup time, CFS; corneal fluorescein staining, BIP; blink interval period.

## Discussion

The new diagnostic criteria for DED have been recommended by ADES, and the diagnosis of DED can be made by subjective symptoms and decreased TFBUT (tear film instability)^19^. In this study, we showed the usefulness of MBI measurement as a simple DED screening method. The evaluation of tear film stability is important in the diagnosis of DED^1,19^, MBI measurement can be useful for many people if it can substitute for the measurement of TFBUT.

Our study demonstrated that MBI is strongly correlated with TFBUT (r = 0.464), indicating that tear breakup stresses the corneal surface, stimulates the underlying nociceptors, and induces blinking. Although MBI is thought to be associated with subjective symptoms, our correlation analysis showed that there was no significant correlation between MBI and subjective symptoms (DEQS), suggesting that MBI is independent from subjective symptoms and reflects the influence of TFBUT. Since the ADES diagnostic criteria are based on TFBUT and subjective symptoms, we concluded that MBI is suitable for the new ADES diagnostic criteria because MBI was associated with TFBUT in this study (Table 2).

Our study demonstrated that MBI was significantly shortened in DED patients compared to non-DED patients, which has also been observed in previous studies^16,17^. We have also suggested the optimal cutoff value of MBI (12.4 seconds) for detecting DED according to the ADES criteria. As shown in Table 3, using a cutoff of 12.4 seconds of MBI for DED screening, the positive predictive value was 76.9% and the positive likelihood ratio was 1.68, indicating that MBI could accurately detect patients with DED. On the other hand, since ADES criteria excluded keratoconjunctival epithelial disorder and Schirmer test for the DED diagnosis^19^, MBI may not be suitable to evaluate keratoconjunctival epithelial disorder and tear-deficient DED, indicating that MBI should not be used to judge the severity or classification of DED.

A variety of assessments have been utilized in evaluating DED, including measurement of TFBUT, CFS, Rose Bengal staining, Schirmer test and osmolality measurement^2^; however, most of these assessments need dedicated reagents and machines. IBI, another evaluation item that is similar to MBI^14^, also decreases in patients with DED; however, self-measurement of IBI is difficult, and the routine blink rate has not been shown to be related to local corneal and conjunctival factors^17^. Since MBI is a noninvasive and simple test, it is useful for self-screening in people with undiagnosed DED, and it offers the opportunity for making an appropriate diagnosis and subsequent proper use of eye drops for DED, including the use of over-the-counter eye drops.

BIP represents the time between the timing of dark spots occurrence on the ocular surface to the blinking (Fig. 1). This study showed that the BIP was reduced in the DED group compared to the one in the non-DED group (Table 1), suggesting that the instability of the tear film layer in DED shortens not only the TFBUT but also the BIP. A previous study reported that the total area of the tear film breakup was increased in DED^23^, via the mechanism of the decreased BIP due to the stimulation of the underlying nociceptors^17^. Therefore, we proposed that the mechanism of reduced both TFBUT and BIP (=MBI) is an increase in the number of blinking in patients with DED. Since it is expected that BIP will decrease in the evaporative-type DED, it may be important to study the BIP depending on the type of DED.

This study has a few limitations. This study may have selection bias because it was conducted in a university hospital in Japan and there are more female participants likely due to the predominance of females affected by DED^24^. We did not exclude patients with systemic diseases and systemic treatments. In addition, we did not measure Rose Bengal stain scores, tear osmolality, meibomian gland dysfunction, and corneal sensations to classify the DED in all participants because we wanted to develop a simple screening test that does not require dedicated reagents and machines.

In summary, the results of this study suggest MBI is useful in screening for DED. Specifically, the MBI of 12.4 seconds or shorter was found to suggest a diagnosis of DED based on the 2016 ADES criteria. MBI may be useful for people with undiagnosed DED, and it offers the opportunity for conducting more specific examinations, such as TFBUT, by ophthalmologists.

## Participants and Methods

### Study design and participants

This cross-sectional observational study included 292 patients recruited between September 2016 to September 2017 at Juntendo University Hospital, Department of Ophthalmology, Tokyo, Japan. A requirement for a written informed consent was waived due to the retrospective observational nature of the study, and it was carried out using the opt-out method on our hospital website. The clinical study was approved by the Juntendo University Hospital, Independent Ethics Committee (approval numbers: 15–185 and 17-088) and adhered to the tenets of the Declaration of Helsinki.

### Exclusion criteria

We excluded patients with a history of eye lid disorder, ptosis, mental disease, Parkinson disease, and any other disease that affects blinking.

### Dry eye disease diagnosis and classification

Both eyes in all patients underwent complete ophthalmic evaluation, including measurement of best-corrected visual acuity, intraocular pressure (IOP), and assessment of subjective symptoms using the DEQS questionnaire^22^. TFBUT, CFS for kerato-conjunctival vital staining and Schirmer test Ι for reflex tear production were assessed in both eyes. Since blinking is affected by the condition of both eyes via the corneal reflex^18^, the lower TFBUT and Schirmer test Ι value data were used, whereas higher values of CFS were used in this study. We diagnosed DED and non-DED using the 2016 ADES criteria^19^. The 2016 criteria make a diagnosis of DED with two positive items, specifically positive subjective symptoms and decreased TFBUT (≤5 seconds). We diagnosed DED if either eye had a diagnosis of DED by the 2016 criteria.

### Environmental conditions

Temperature and humidity of the examination room were controlled at 26 °C in the summer and 24 °C in the winter and 50% relative humidity, according to the Guideline for Design and Operation of Hospital HVAC Systems established by Healthcare Engineering Association of Japan Standard^25^.

### Subjective symptoms and Dry Eye-Related Quality-of-Life Score (DEQS)

Subjective symptoms were assessed by interviewing participants with DED. The DEQS questionnaire was administered to assess the severity of dry eye-associated symptoms and the multifaceted effects of DED on the patients’ daily lives^22^. The score derived from this questionnaire is a subjective measurement of DED symptoms where 0 indicates the best score (no symptoms) and 100 indicates the worst score (maximum symptoms).

### Clinical assessments for DED

TFBUT and kerato-conjunctival vital staining (CFS) were assessed with fluorescein sodium (Fluores Ocular Examination Test Paper, Ayumi Pharmaceutical Co., Tokyo, Japan) staining. We performed TFBUT, CFS, MBI measurement, and Schirmer test I.

### Tear film breakup time (TFBUT)

TFBUT was measured using a fluorescein dye according to the standard method^19^. To minimize the effect of the test strip on tear volume and TFBUT, a small quantity of the dye was administered with a wetted fluorescein strip. After the dye was instilled, the subject was instructed to blink three times to ensure adequate mixing of the dye with the tears. The time interval between the last blink and the appearance of the first dark spot on the cornea was measured with a stopwatch. The mean value of three measurements was used. The cutoff value of TFBUT ≤5 seconds was used to diagnose DED^19^.

### Kerato-conjunctival vital staining (cornea fluorescence staining [CFS])

CFS was graded according to the van Bijsterveld grading system^26^, dividing the ocular surface into three zones: nasal bulbar conjunctiva, temporal bulbar conjunctiva, and cornea. Each zone was evaluated on a scale of 0 to 3, with 0 indicating no staining and 3 indicating confluent staining. The maximum possible score is 9.

### Maximum blink interval (MBI) and blink induction period (BIP)

**Figure 2:**
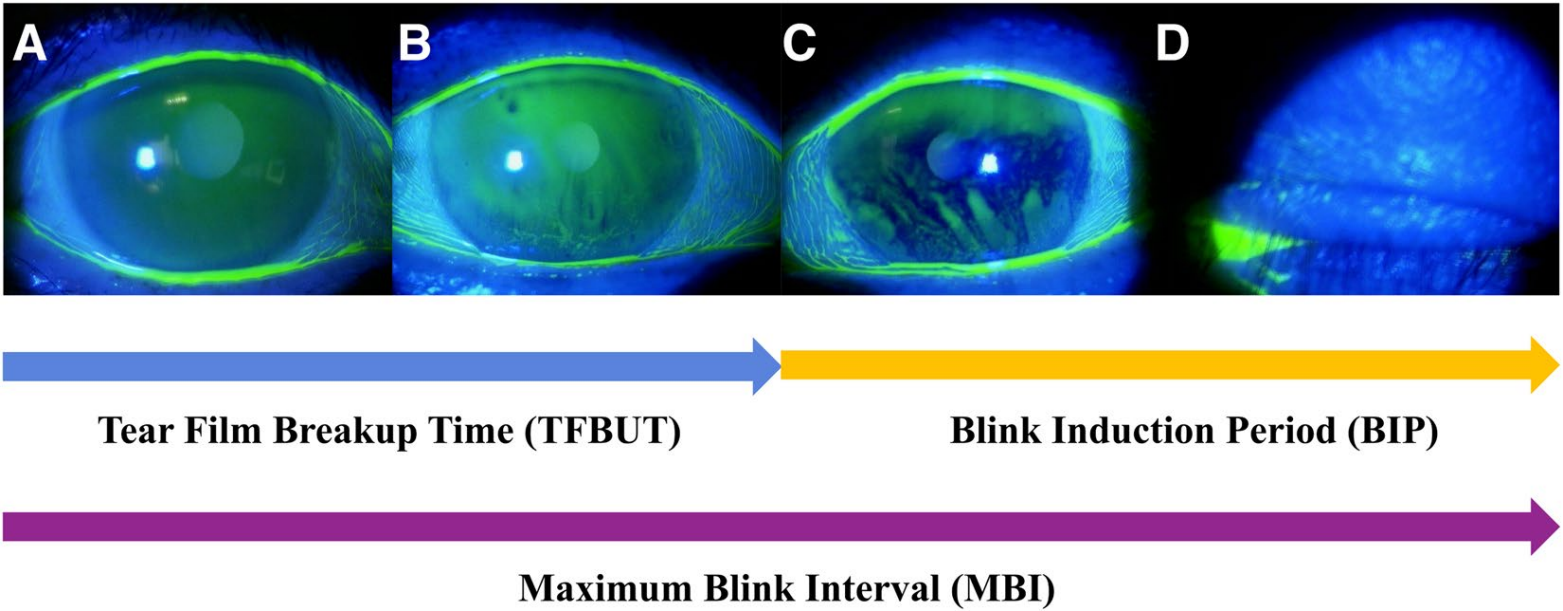
The definition of maximum blink interval (MBI) and blink induction period (BIP). (**A**) Show how to determine the tear film breakup time (TFBUT), MBI and BIP. TFBUT was the time interval between the last blink (**A**) and the appearance of the first dark spot on the cornea (**B**). MBI was the length of time that participants could keep the eye open before blinking (**A–D**). BIP was calculated by subtracting TFBUT from MBI (**C,D**). MBI; maximum blink interval, TFBUT; tear film breakup time, BIP; blink interval period.

The length of time that the participants could keep the eye open before blinking during each trial was termed the MBI (Fig. 2). We measured the MBI twice by a stopwatch under a light microscope without light. MBI was recorded as 30 seconds if it exceeded 30 seconds. BIP was calculated by subtracting TFBUT from MBI.

### Schirmer test I

The Schirmer test I was performed without topical anesthesia after the completion of all other examinations. Schirmer test strips (Ayumi Pharmaceutical Co., Tokyo, Japan) were placed at the outer one-third of the temporal lower conjunctival fornix for 5 minutes. The strips were then removed, and the length of dampened filter paper (in mm) was recorded.

### Statistical analyses

To compare the characteristics of study participants, the *t* test was used for continuous variables and χ^2^ test was used for categorical variables. Pearson correlation coefficients were estimated among MBI, DEQS, TFBUT, and Schirmer test Ι . A ROC analysis was conducted to examine the diagnostic efficacy of MBI for DED. The ROC curve was plotted by computing the sensitivity and specificity using each symmetric value of the rating variable as a possible cutoff point. A point was plotted on the graph for each of the cutoff points; these plotted points were joined by straight lines to form the ROC curve, and the AUC was estimated using the trapezoidal rule. To determine the optimal cutoff value of MBI for detecting DED, the point where sensitivity and specificity were maximized was identified. Data are presented as means ± standard deviations (SDs) or proportions. Statistical analyses were performed using STATA version 14 (Stata Corp, Texas). A *P* < 0.05 was considered significant.
